# A New Sanitarium in Austin

**Published:** 1917-04

**Authors:** 


### A New Sanitarium in Austin.

Just one mile from the city limits, in the very heart of a six-acre plot, surrounded by about four hundred stately elms and oaks, is the new Oaks Sanitarium.

Dr. Ralph E. loud, formerly of the Heights Sanitarium of Houston, is the proud owner of this new sanitarium. The editor feels that Dr. Cloud has a perfect right to be proud of this place, for it is an ideal location for a sanitarium that makes a specialty of nervous and mental diseases.

Far away from the noise and dust and the hustle and bustle of the city, but with the city's conveniences of water, electric lights, steam heat, telephone, baths, conservatory, library, rest rooms, parlors, the patient will find everything for his comfort and all the modern conveniences of any city hospital.

There are thirty-five acres of good rich land surrounding the Sanitarium, and Dr. Cloud will grow his own gardens, raise his own chickens, and furnish the dairy products for his institution. This is quite an advantage, as he will then be assured of the freshness and purity of these most important of all articles of diet.

At present Dr. Cloud has a capacity for fifteen patients, but in the very near future he expects to build a separate hall for the male patients, which will more than double the present capacity.

This is not new work for the Doctor. For more than ten years he has devoted himself exclusively to the treatment of nervous and mental diseases, and his experience in the insane asylum in San Antonio and Terrell, together with five years of private practice in Houston, has ably fitted him for this kind of work.

Assisted by experienced nurses, Dr. Cloud gives his personal attention to every patient entering the Sanitarium.

The physicians of Austin and the surrounding country have long felt the need of such an institution as this near at hand, and they are taking quite an interest and local pride in the new Oaks Sanitarium.

